# Linking salinity stress tolerance with tissue-specific Na^+^ sequestration in wheat roots

**DOI:** 10.3389/fpls.2015.00071

**Published:** 2015-02-20

**Authors:** Honghong Wu, Lana Shabala, Xiaohui Liu, Elisa Azzarello, Meixue Zhou, Camilla Pandolfi, Zhong-Hua Chen, Jayakumar Bose, Stefano Mancuso, Sergey Shabala

**Affiliations:** ^1^Faculty of Science, Engineering and Technology, School of Land and Food, University of TasmaniaHobart, TAS, Australia; ^2^School of Science and Health, University of Western SydneySydney, NSW, Australia; ^3^School of Chemical Engineering and Technology, Tianjin UniversityTianjin, China; ^4^Department of Agrifood Production and Environmental Sciences, University of FlorenceFlorence, Italy

**Keywords:** bread wheat, cytosolic Na^+^, Na^+^ distribution, root zones, salinity stress tolerance, vacuolar Na^+^ sequestration

## Abstract

Salinity stress tolerance is a physiologically complex trait that is conferred by the large array of interacting mechanisms. Among these, vacuolar Na^+^ sequestration has always been considered as one of the key components differentiating between sensitive and tolerant species and genotypes. However, vacuolar Na^+^ sequestration has been rarely considered in the context of the tissue-specific expression and regulation of appropriate transporters contributing to Na^+^ removal from the cytosol. In this work, six bread wheat varieties contrasting in their salinity tolerance (three tolerant and three sensitive) were used to understand the essentiality of vacuolar Na^+^ sequestration between functionally different root tissues, and link it with the overall salinity stress tolerance in this species. Roots of 4-day old wheat seedlings were treated with 100 mM NaCl for 3 days, and then Na^+^ distribution between cytosol and vacuole was quantified by CoroNa Green fluorescent dye imaging. Our major observations were as follows: (1) salinity stress tolerance correlated positively with vacuolar Na^+^ sequestration ability in the mature root zone but not in the root apex; (2) contrary to expectations, cytosolic Na^+^ levels in root meristem were significantly higher in salt tolerant than sensitive group, while vacuolar Na^+^ levels showed an opposite trend. These results are interpreted as meristem cells playing a role of the “salt sensor;” (3) no significant difference in the vacuolar Na^+^ sequestration ability was found between sensitive and tolerant groups in either transition or elongation zones; (4) the overall Na^+^ accumulation was highest in the elongation zone, suggesting its role in osmotic adjustment and turgor maintenance required to drive root expansion growth. Overall, the reported results suggest high tissue-specificity of Na^+^ uptake, signaling, and sequestration in wheat roots. The implications of these findings for plant breeding for salinity stress tolerance are discussed.

## Introduction

More than 800 million hectares (6%) of land are affected by salinity worldwide (Munns and Tester, 2008). As sodium is one of the most abundant metal elements, sodium salts dominate in many saline soils of the world (Rengasamy, 2006). High concentrations of salts in soils account for large decreases in the yields of a wide variety of crops all over the world (Tester and Davenport, 2003). In the light of predicted population growth to 9.3 billion by 2050 (Lee, 2011), global food requirements are expected to increase by 70–110% (Tilman et al., 2011). Wheat is one of the most important crops providing nearly 55% of the consumed carbohydrates world widely (Gupta et al., 1999) but is not highly salt tolerant, and its commercial production is substantially reduced as the soil salinity level rises to 100 mM NaCl and is not possible in soils containing more than 250 mM NaCl (Munns et al., 2006; Munns and Tester, 2008). Thus, improving salinity stress tolerance in wheat is an urgent task to cope with the possible shortage of food supply in the near future. This is especially true for the hexaploid bread wheat that composes about 95% of all wheat grown world wide (Shewry, 2009).

Sodium uptake and sequestration has always been in spotlight of researchers aimed at finding the traits or genes which can be selected to improving salinity tolerance in wheat. Early studies using ^22^Na^+^ isotopes showed that salt tolerant wheat varieties have significantly lower Na^+^ accumulation in the shoot (Davenport et al., 1997), suggesting an efficient Na^+^ exclusion mechanism. The following studies by Munns and colleagues (Munns et al., 2002, 2003, 2006; Munns and James, 2003; Lindsay et al., 2004) suggested that targeting Na^+^ exclusion from shoot was a promising way to improving salinity tolerance in this species. Indeed, under saline condition, flag leaf Na^+^ was significantly reduced from 326 mM in commercial variety Tomaroi to 87 mM in transgenic Tamaroi plants that expressed *TmHKT1;5-A* gene enabling Na^+^-retrieval from the xylem (Munns et al., 2012). This has resulted in about 20% increase in wheat yield under saline field conditions (from 1.30 to 1.61 tons per hectare). Using microelectrode ion flux measuring MIFE technique, Cuin et al. (2011) found that Kharchia 65 (accepted as a “standard” for salinity tolerance in wheat by most breeders) had the highest root Na^+^ exclusion ability compared with other seven wheat varieties studied. Pharmacological experiments and experiments with transgenic Arabidopsis mutants have confirmed that this Na^+^ efflux was mediated by the plasma membrane SOS1 Na^+^/H^+^ antiporter. Similar studies conducted on sorghum (Yang et al., 1990), maize (Fortmeier and Schubert, 1995), and tomato (Al-Karaki, 2000) have also suggested that plant's ability to exclude Na^+^ from uptake in these species was positively correlated with the overall salinity tolerance. Even in lower plants the ability to avoid the accumulation of Na^+^ in cytosol is critical for its salinity tolerance (e.g., cyanobacteria; Allakhverdiev et al., 2000; Allakhverdiev and Murata, 2008).

The above beneficial effects of Na^+^ exclusion from uptake was always attributed to its toxic effect on cell metabolism (Maathuis and Amtmann, 1999; Munns and Tester, 2008) and essentiality to maintain low level of Na^+^ in the cytosol. However, the same goal may be achieved by the efficient Na^+^ sequestration in the vacuole. The latter trait is commonly employed by halophytes (naturally salt-loving plants; Flowers and Colmer, 2008; Shabala and Mackay, 2011; Shabala, 2013; Bonales-Alatorre et al., 2013a,b), and some evidences were presented that salt-tolerant wheat varieties may also possess better vacuolar Na^+^ sequestration ability (e.g., Saqib et al., 2005). However, most of these studies were conducted on leaves, while the role of Na^+^ sequestration in roots received less attention. In *Thellungiella salsuginea*, a halophytic relative of *Arabidopsis thaliana*, Oh et al. (2009) showed that vacuolar Na^+^ fluorescence intensities in cortex cells of root tip region is higher in *thsos1-4* than in wild type. However, to the best of our knowledge, the issue of tissue-specificity of vacuolar Na^+^ sequestration between different root zones, and its link with the overall salinity tolerance, has never received a proper attention, neither in wheat nor in any other crop species.

The root anatomy and functional structure can be generally divided into four different zones: (1) root meristem, (2) the distal elongation (or transition) zone, (3) elongation zone, and (4) mature zone (Verbelen et al., 2006; Baluška and Mancuso, 2013). So far, most studies of Na^+^ distribution in plant roots under salt stress was conducted either at the level of whole root (e.g., Matsushita and Matoh, 1991; Flowers and Hajibagheri, 2001; Rus et al., 2001), or were focused on cell-type-specific Na^+^ distribution in roots (Huang and van Steveninck, 1988; Storey et al., 2003; Oh et al., 2009, 2010). While these and some other (Cuin et al., 2011; Li et al., 2012) papers showed a heterogeneity of Na^+^ distribution within the root, none of them discussed the difference in Na^+^ patterning between intracellular compartments within functionally different root zones. In *T. salsuginea*, Na^+^ accumulated inside the pericycle in *thsos1-4* mutant, while in the wild type it was confined in vacuoles of epidermal and cortical cells (Oh et al., 2009). Cell-type-specific Na^+^ distribution patterns in hypodermis, cortex, endodermis, and pericycle were also studied in salinized grapevines using X-ray microscopy method (Storey et al., 2003). Here, vacuolar Na^+^ was sequestered predominantly in endodermis and pericycle cells. However, to the best of our knowledge, no study has compared Na^+^ distribution between cytosol and vacuole in functionally different root zones within the same tissue, at least in bread wheat.

In the present work, variability of vacuolar Na^+^ sequestration in four different root zones under salt stress was studied using six bread wheat varieties contrasting in their salinity tolerance. Cytosolic and vacuolar Na^+^ content in different root zones was quantified by CoroNa Green fluorescent dye imaging, and the link between tissue-specific vacuolar Na^+^ sequestrations in specific root zones and the overall salinity stress tolerance was explored. We report that the overall salinity stress tolerance correlates positively with vacuolar Na^+^ sequestration ability in the mature root zone but not in the root apex. At the same time, cytosolic Na^+^ levels in root meristem were significantly higher in salt tolerant than sensitive group, suggesting that meristem cells may play a role of the “salt sensor.” The overall Na^+^ accumulation was highest in the elongation zone, suggesting its role in osmotic adjustment and turgor maintenance required to drive root expansion growth.

## Materials and methods

### Plant materials and growth conditions

Six bread wheat (*Triticum aestivum*) varieties contrasting in their salinity tolerance (tolerant – Persia 118, Cranbrook, and Westonia; sensitive – Iran 118, Belgrade 3, and 340) were used in this study. All seeds were obtained from the Australian Winter Cereals Collection and multiplied in our laboratory. Plants were grown in February–March 2013 in the glasshouse facilities at the University of Tasmania essentially as described in Chen et al. (2007b). Twelve seeds for each variety were sown in 4.5 L PVC pots with the standard potting mix by triplicates. After emerging (roughly 6 days), salt treatment (300 mM NaCl) were applied for about 5 weeks. Plants were irrigated twice per day by an automatic watering system with dripper outlets, and were uniformly thinned to eight plants in each pot after roughly 10 days sowing. A saucer was placed under each pot. For confocal imaging experiments, seeds were sterilized with 5% commercial bleach for 15 min, and then washed thoroughly by the running tap water for half an hour. Seeds were then germinated in wet paper rolls in growth chambers at 23 ± 1°C (16 h light/8 h dark regime). Four days old wheat roots were treated with 100 mM NaCl for 72 h, and then stained with CoroNa Green dye for the LSCM (laser scanning confocal microscopy) measurements as described below.

### Whole-plant performance assessing

Before harvesting, plants “damage index” were scored on zero to 10 scale (0, no visual symptoms of stress; 10, dead plants; Supplementary Figure S1). The higher damage index score represents the lower salt tolerance. Then, the stem was cut 1 cm above the ground, and shoot fresh weight (FW) was measured.

### Confocal laser scanning microscopy measurements

**Figure 1:**
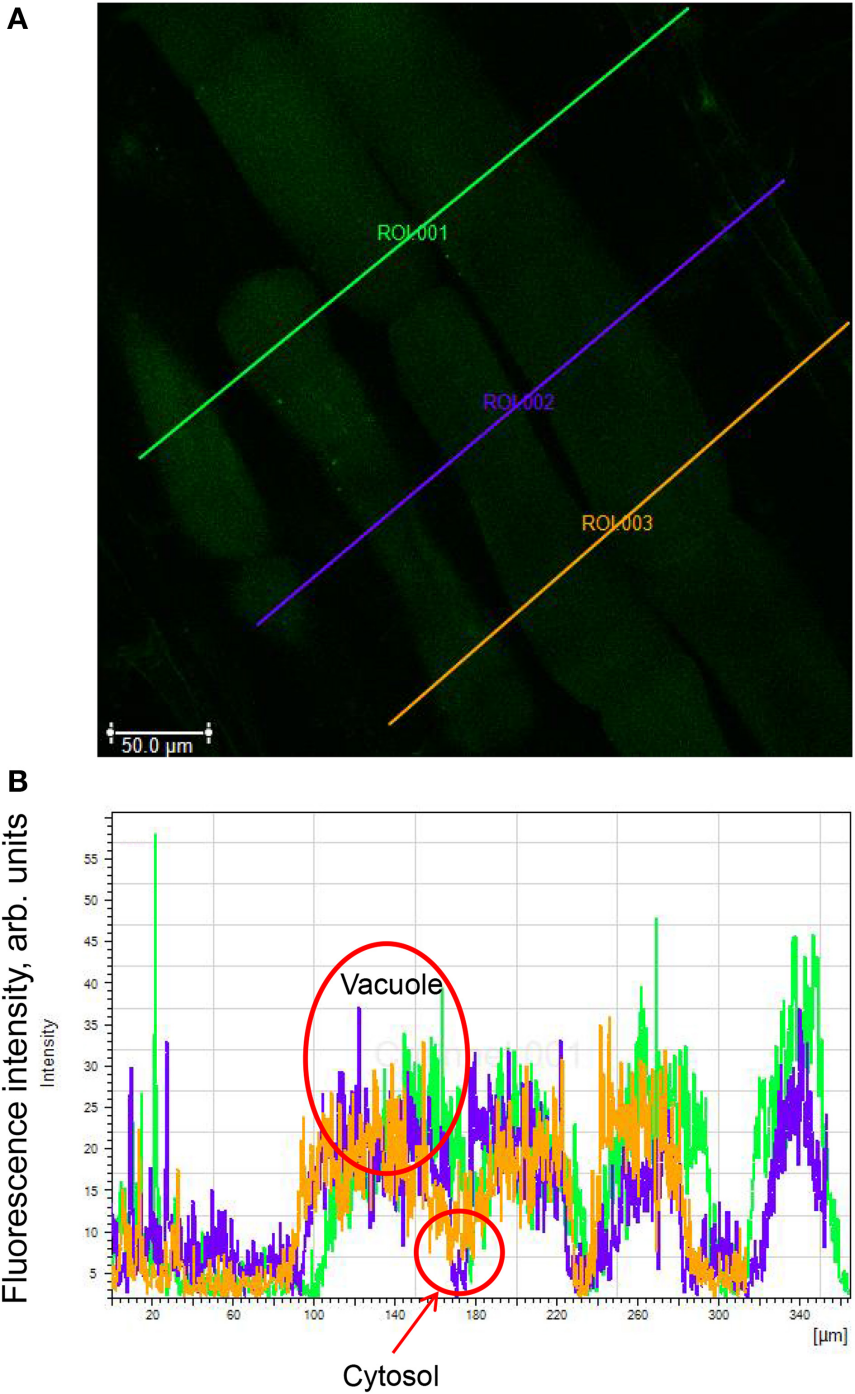
**Illustration of the quantification procedure for Na^+^ distribution between the cytosol and the vacuole**. **(A)** Several lines are drawn across the so-called “region of interest” (ROI) in an appropriate root zone. **(B)** Continuous fluorescence intensity distribution profiles obtained by LAS software. The mean fluorescence intensity values for cytosolic and vacuolar compartments are then calculated for each cell by attributing signal profiles to root morphology (visualized by light microscopy images; not shown in a figure). The shown image was obtained from mature region of Persia 118 cultivar.

Measurements of cytosolic and vacuolar Na^+^ content in wheat root cells using the green fluorescent Na^+^ dye CoroNa Green acetoxymethyl ester were essentially as described in Bonales-Alatorre et al. (2013b). The dye has absorption and fluorescence emission maxima of approximately 492 and 516 nm, respectively. The dye was reconstituted as a stock with anhydrous dimethyl sulfoxide before use. The CoroNa Green indicator stock was added to 5 mL of measuring buffer (10 mM KCl, 5 mM Ca^2+^-MES, pH 6.1) and diluted to a final concentration of 15 mM. Ten millimeters-long root segments were cut from the apical (the first 10 mm from the apex) and mature (30–40 mm from the apex) root zones and incubated for 2 h in the dark in a solution containing 20 μM CoroNa Green. After incubation, the samples were rinsed in a buffered MES solution and examined using confocal microscopy. Confocal imaging was performed using an upright Leica Laser Scanning Confocal Microscope SP5 (Leica Microsystems, Germany) equipped with a 40× oil immersion objective. To analyze sodium intracellular localisation CoronaNa Green AM (Molecular Probes, USA) was used. The excitation wavelength was set at 488 nm, and the emission was detected at 510–520 nm. Comparison of different levels of fluorescence between cells was carried out by visualizing cells with the identical imaging settings of the confocal microscope (i.e., exposure times, laser intensity, pinhole diameter and settings of the imaging detectors). Images were analyzed with LAS Lite software. Six to eight individual roots (each from different plants) were used; and at least two images were taken for each root zone. For analysis, several lines were drawn across the so-called “region of interest” (ROI; Figure 1A) in an appropriate root zone. Continuous fluorescence intensity distribution profiles (quantified in arbitrary units by LAS software) were then obtained and plotted in an Excel file (Figure 1B). The mean fluorescence intensity values for cytosolic and vacuolar compartments were then calculated for each cell by attributing signal profiles to root morphology (visualized by light microscopy images). The data was then averaged for all cells measured for the same treatment. The background signal was measured from the empty region and then subtracted from the readings, to obtain corrected fluorescence values. Depending on the root zone, readings from between 70 and 300 cells were averaged and reported for each genotype.

**Figure 2:**
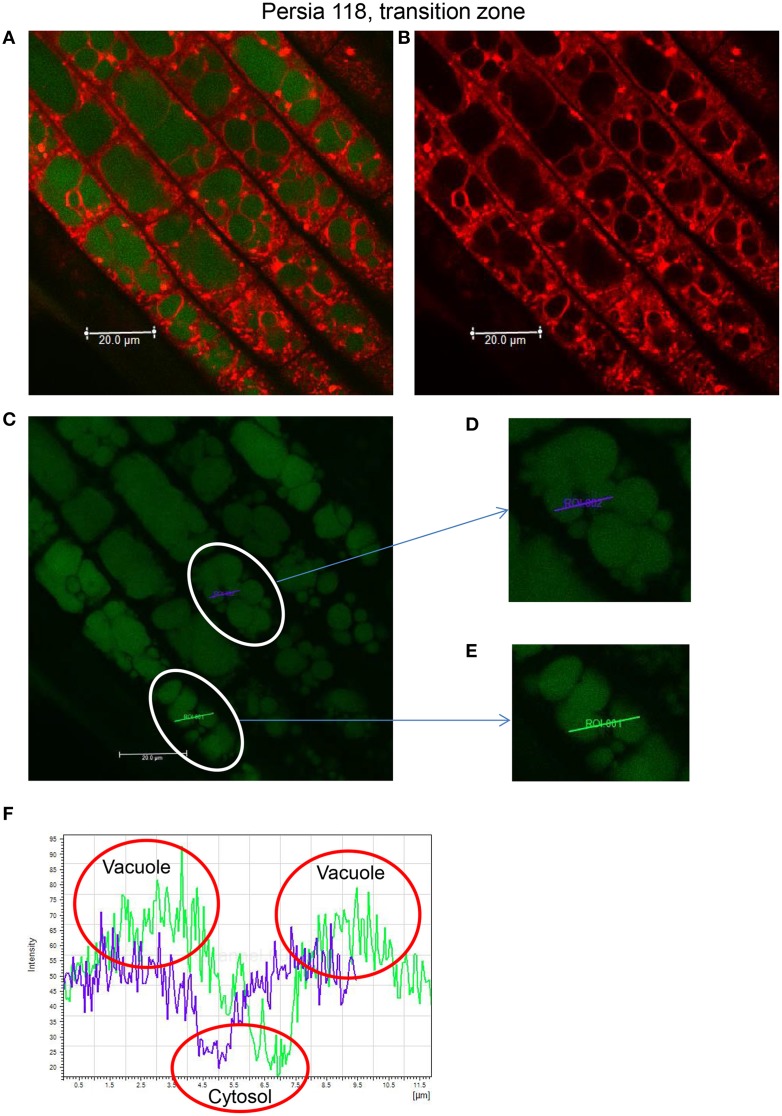
**Quantifying intracellular Na^+^ distribution between the cytosol and the vacuole by double staining procedure**. Intracellular Na^+^ distribution is illustrated using the transition zone of cultivar Persia 118 as an example. **(A)** The root transition zone co-stained with Corona Green AM and FM4-64. The same root stained with FM4-64 dye **(B)** and CoroNa Green **(C)**. Two cells having multiple vacuoles (circled) were then selected for analysis (depicted in **D**,**E**). Two lines were then drawn across the “region of interest” (ROI) for each of these, crossing two vacuoles in each of cells. Intracellular Na^+^ distribution was then quantified **(F)**.

A further validation of the above protocol was conducted using roots co-stained with CoroNa Green-AM and FM4-64, a dye that stains both plasma and vacuolar membranes (Oh et al., 2010; Bassil et al., 2011) and allow a better resolution between intracellular compartments. After 1 h incubation with 20 μM CoroNa Green-AM (as described above), the same root samples were then incubated together with 20 μM FM4-64 for another 1 h to visualize tonoplast. Roots were then rinsed with buffer solution (10 mM KCl, 5 mM Ca^2+^-MES, pH 6.1) for 3 min and analyzed using confocal imaging facilities. For FM4-64 fluorescence, the 488-nm excitation line was used and collected with a 615-nm long-pass filter. Results of this experiment are illustrated in Figure 2 showing intracellular Na^+^ distribution in the transition zone of cultivar Persia 118. Figure 2A shows distribution of CoroNa Green and FM4-64 in co-stained root samples, while Figures 2B,C show the same root stained with FM4-64 and CoroNa Green, respectively. Two cells having multiple vacuoles (circled) were then selected for analysis (depicted in Figures 2D,E). Two ROI lines were then drawn crossing two vacuoles in each of the cells. Intracellular Na^+^ distribution was then quantified (Figure 2F). As one can see, two major peaks in each cell correspond to two vacuoles, while troughs report values for cytosolic Na^+^.

### Statistical analysis

All data were analyzed by using SPSS 20.0 for windows (SPSS Inc., Chicago, IL, USA). Comparison of cytosolic or vacuolar Na^+^ fluorescent intensity between different varieties in root zones was done by One-Way ANOVA based on Duncan's multiple range test. Different lowercase letters represent significant difference between varieties. Data with the same lowercase letters are not significantly different at *P* < 0.05. Comparison of cytosolic or vacuolar Na^+^ fluorescent intensity between tolerant and sensitive groups in different root zones was done by independent samples *t*-test. The significance levels are ^*^P < 0.05, ^**^P < 0.01, and ^***^P < 0.001.

## Results

### Whole-plant performance

**Figure 3:**
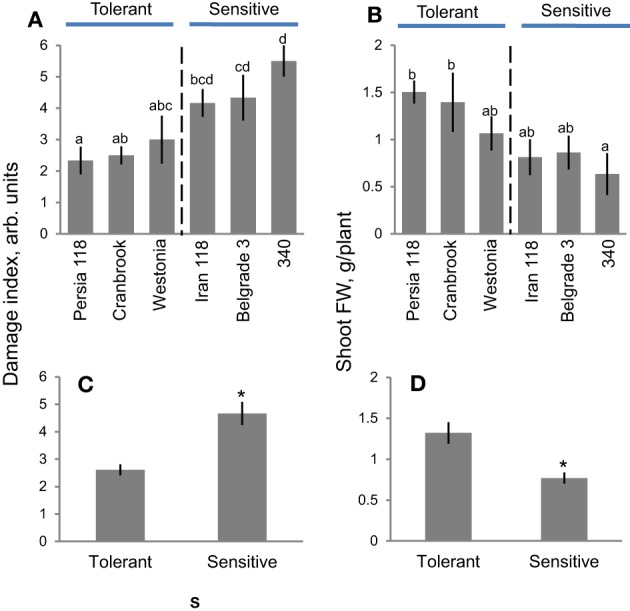
**Genetic variability of salinity stress tolerance in bread wheat**. Damage index **(A)** and shoot fresh weight **(B)** of **six** bread wheat cultivars contrasting in their salinity tolerance after 38 days of 300 mM NaCl treatment in a glasshouse. Different lowercase letters represent significant differences between varieties at *P* < 0.05. **(C,D)** Average pooled data for tolerant and sensitive clusters shown in **A,B**. Mean ± SE (*n* = 3; 24 plants in total, eight plants in each pots). Asterisk indicates significant difference between clusters at *P* < 0.05.

Bread wheat varieties used in this study showed big variability in salinity stress tolerance. Salinity damage index (a measure of salt tolerance; see Supplementary Figures S1A,B for details) ranged from the highest (most sensitive) 5.5 ± 0.5 in variety 340 to the lowest (most tolerant) 2.3 ± 0.4 in variety Persia 118 (significant at *P* < 0.01; Figure 3A). Similarly, the highest shoot fresh weight (FW) was found in Persia 118 (1.5 ± 0.1 g/plant), while variety 340 showed the lowest shoot FW (0.6 ± 0.2 g/plant) (Figure 3B). Accordingly, all varieties were grouped into tolerant and sensitive clusters (Figures 3C,D). The tolerant cluster showed much less damage (about two-fold; *P* < 0.05, Figure 3C) and 70% higher shoot biomass (significant at *P* < 0.05, Figure 3D) compared with the sensitive cluster. A significant negative correlation (*r*^2^ = 0.91, *P* < 0.01) was found between shoot FW and damage index among all the varieties (Supplementary Figure S1C).

### Sodium accumulation profiles

**Figure 4:**
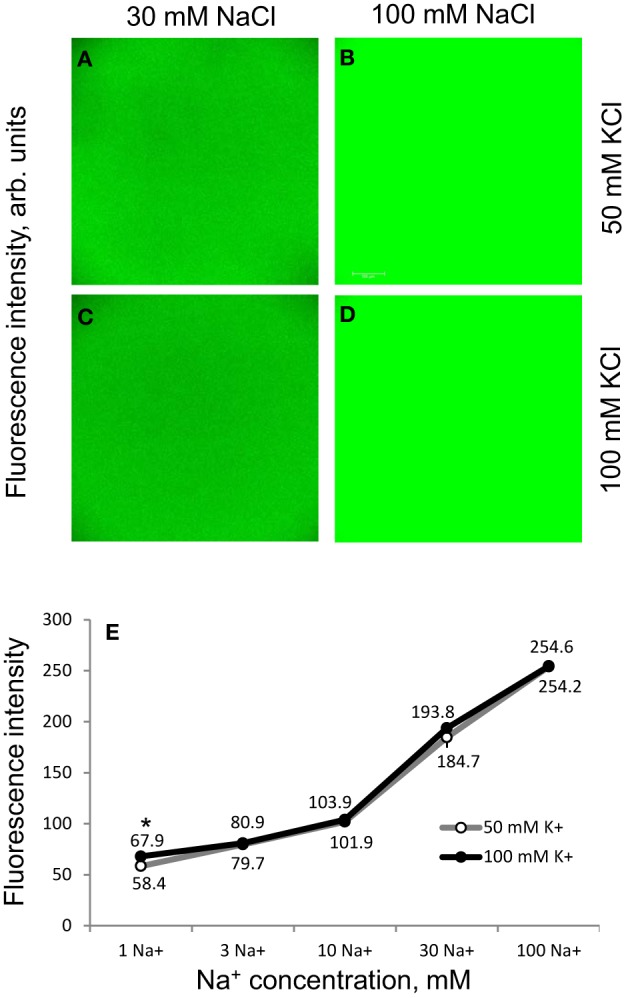
**Methodological aspects of CoroNa Green dye calibration**. CoroNa Green dye was calibrated *in vitro* in a buffer solution containing different Na^+^ and K^+^ concentrations. **(A–D)** *In vitro* fluorescence images for two Na^+^ and two K^+^ concentrations used in experiment. One (of four) representative images is shown for each Na^+^ and K^+^ concentration; **(E)** dose-dependency of Na^+^ fluorescence signals at 50 and 100 mM KCl background. Mean ± SE (*n* = 60; four images, 15 replicates recorded from each image). Asterisk indicates a significant (at *P* < 0.05) difference between K^+^ treatments. Please note that in most cases the error bar is smaller than the symbol *per se*.

Before cell- and genotype-specific Na^+^ distribution was quantified, a series of methodological experiments was conducted to eliminate possible confounding effects of dye loading and stress-induced changes in intracellular ionic conditions on fluorescence measurements. First, CoroNa Green was calibrated in a cytosol-like solution (50–100 mM K^+^; <1 μM Ca^2+^; pH = 7.2) in a broad range of Na^+^ concentrations (1, 3, 10, 30, and 100 mM) in *in vitro* experiments. As shown in Figure 4, a two-fold drop in background K^+^ concentration from 100 to 50 mM (mimicking NaCl-induced reduction in cytosolic K^+^ under stress conditions; Shabala et al., 2006) had no significant (*P* < 0.05) impact on fluorescence signal except the lowest (1 mM NaCl) concentration, which is well-below expected levels for cytosolic Na^+^ (Munns and Tester, 2008). Calibration characteristics were also insensitive to pH in physiological (5–7.2) pH range (data not shown). Thus, the possible difference in K^+^ retention ability or stress-induced changes in intracellular pH between genotypes had no confounding effects on CoroNa Green readings. Dye loading profiles were uniform between various Z-plains (Supplementary Figure S2) suggesting that 2 h of loading was sufficient to ensure its homogenous uptake by most cells.

**Figure 5:**
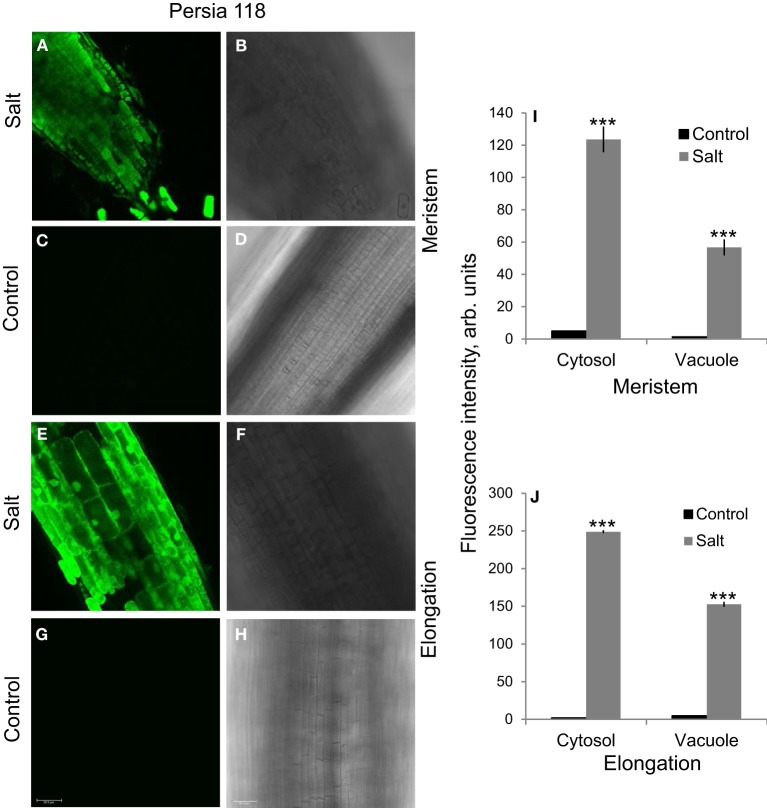
**The comparison of Na^+^ fluorescence intensity in salt-grown and Na-free roots. (A,C,E,G)** Na^+^ fluorescence intensity was measured in root of Persia 118 cultivar grown in the presence (100 mM NaCl for 72 h) and absence (milliQ water) of Na^+^ in the growth media. One (of 12) representative images is shown for each zone. **(B,D,F,H)** Light images on the appropriate slides shown on the left. **(I,J)** Quantification of cytosolic and vacuolar Na^+^ in salt-grown and Na-free roots. Mean ± SE (*n* = 42–96 cells from six roots). ^***^indicates significant differences at *P* < 0.001.

Plants grown in Na-free solution (Milli-Q water) showed negligible small Na^+^ fluorescence signals (Figure 5) while root exposure to 100 mM NaCl for 72 h resulted in massive accumulation of Na^+^ in root tissues (Figure 5).

**Figure 6:**
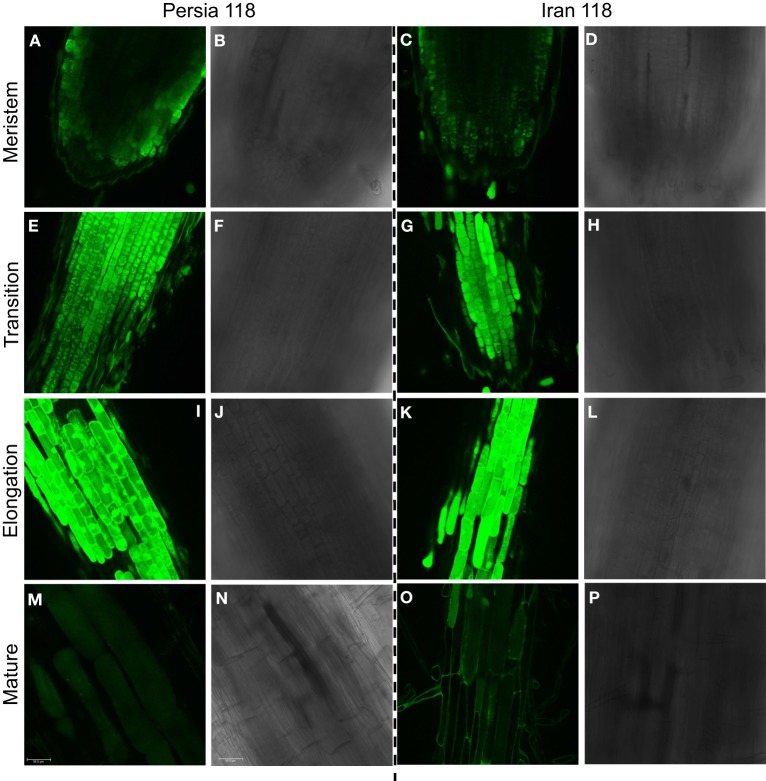
**Na^+^ accumulation profiles in bread wheat visualized by CoroNa Green dye imaging**. The representative images of Na^+^ distribution within one tolerant (Persia 118) and one sensitive (Iran 118) varieties are shown for four different root zones: meristem **(A,C)**; transition zone **(E,G)**; elongation zone **(I,K)**, and mature zone **(M,O)**. One of 12 representative images is shown for each zone. Panels next to fluorescence images represent respective light images **(B,D,F,H,J,L,N,P)**.

**Figure 7:**
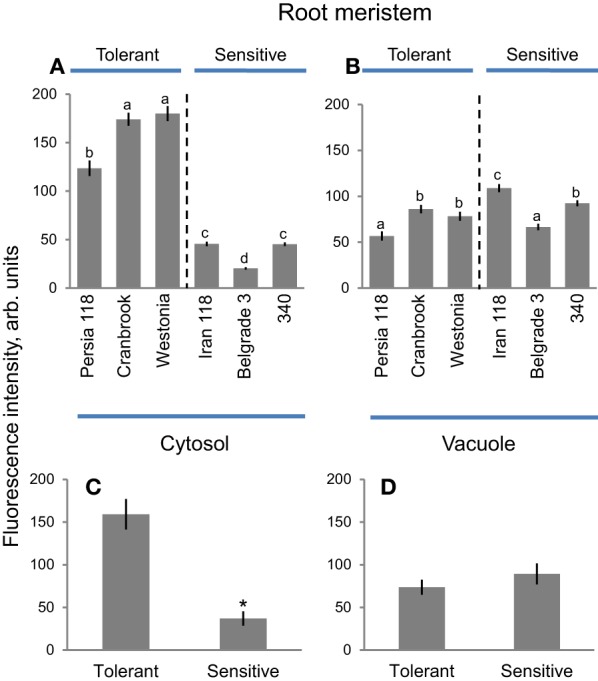
**Na^+^ accumulation and patterning between cytosol and vacuole in meristematic root zone in bread wheat genotypes**. Intensity of CoroNa Green fluorescence in cytosolic **(A)** and vacuolar **(B)** compartments (arb. units) in root meristem of six bread wheat varieties contrasting in their salinity tolerance. Mean ± SE (*n* = 72–96 cells from at least six individual plants). Different lowercase letters represents significant difference between varieties at *P* < 0.05. **(C,D)** Averaged pooled values for cytosolic **(C)** and vacuolar **(D)** Na^+^ intensity for salt-tolerant and salt-sensitive clusters shown in **A**,**B**. Mean ± SE (*n* = 215 to 300; three varieties × 72–96 cells analyzed for each variety). Asterisk indicates significant difference between clusters at *P* < 0.01.

**Figure 8:**
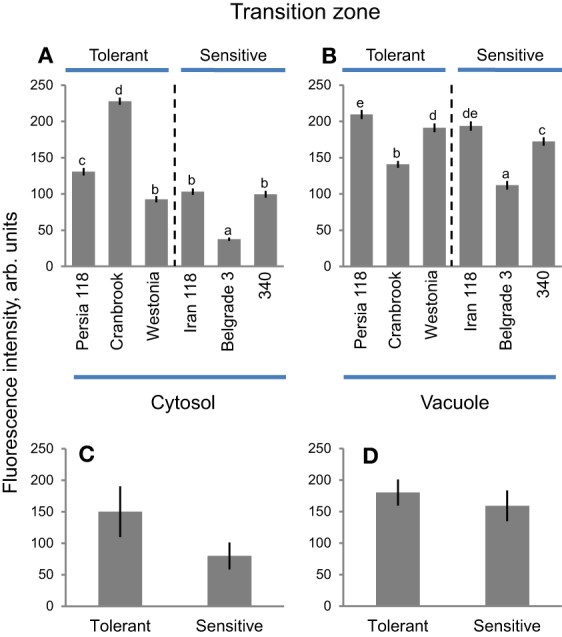
**Na^+^ accumulation and patterning between cytosol and vacuole in transition root zone in bread wheat genotypes**. Intensity of CoroNa Green fluorescence in cytosolic **(A)** and vacuolar **(B)** compartments (arb. units) in transition root zone of six bread wheat varieties contrasting in their salinity tolerance. Mean ± SE (*n* = 72–96 cells from at least six individual plants). Different lowercase letters represents significant difference between varieties at *P* < 0.05. **(C, D)** Averaged pooled values for cytosolic **(C)** and vacuolar **(D)** Na^+^ intensity for salt-tolerant and salt-sensitive clusters shown in **A**,**B**. Mean ± SE (*n* = 215 to 300; three varieties × 72–96 cells analyzed for each variety).

**Figure 9:**
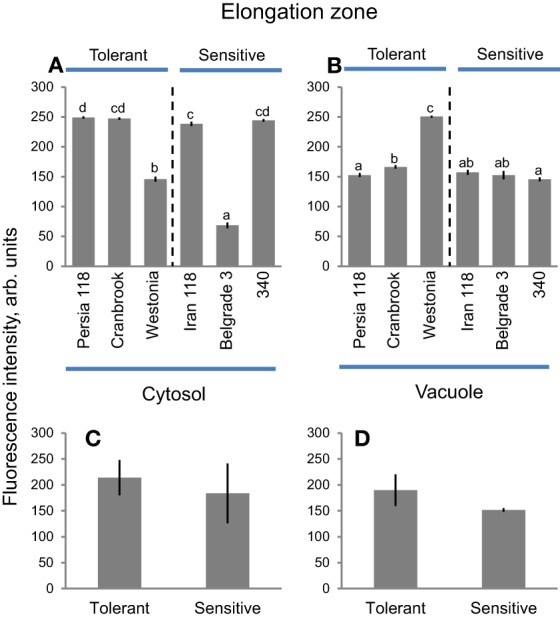
**Na^+^ accumulation and patterning between cytosol and vacuole in elongation root zone in bread wheat genotypes**. Intensity of CoroNa Green fluorescence in cytosolic **(A)** and vacuolar **(B)** compartments (arb. units) in root elongation zone of six bread wheat varieties contrasting in their salinity tolerance. Mean ± SE (*n* = 72–96 cells from at least six individual plants). Different lowercase letters represents significant difference between varieties at *P* < 0.05. **(C,D)** averaged pooled values for cytosolic **(C)** and vacuolar **(D)** Na^+^ intensity for salt-tolerant and salt-sensitive clusters shown in **A,B**. Mean ± SE (*n* = 215–300; three varieties × 72–96 cells analyzed for each variety).

**Figure 10:**
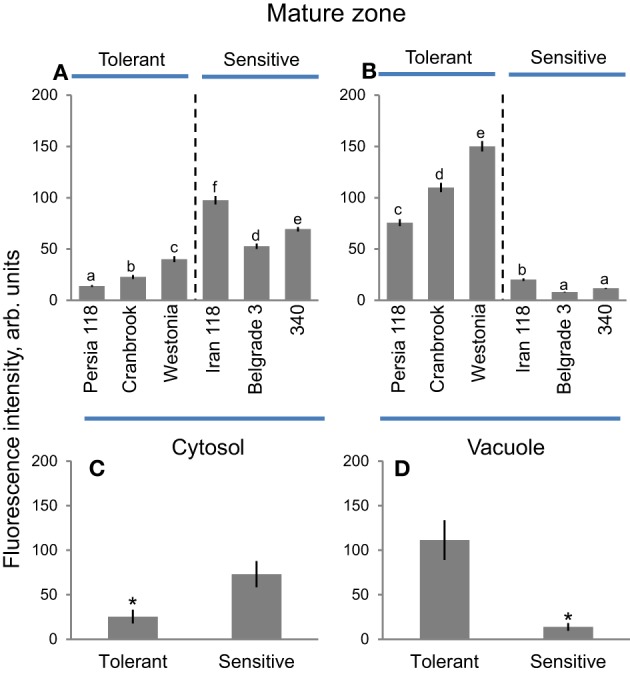
**Na^+^ accumulation and patterning between cytosol and vacuole in mature root zone in bread wheat genotypes**. Intensity of CoroNa Green fluorescence in cytosolic **(A)** and vacuolar **(B)** compartments (arb. units) in mature root zone of six bread wheat varieties contrasting in their salinity tolerance. Mean ± SE (*n* = 72–96 cells from at least six individual plants). Different lowercase letters represents significant difference between varieties at *P* < 0.05. **(C,D)** Averaged pooled values for cytosolic **(C)** and vacuolar **(D)** Na^+^ intensity for salt-tolerant and salt-sensitive clusters shown in **A**, **B**. Mean ± SE (*n* = 240–320; three varieties × 72–96 cells analyzed for each variety). Asterisk indicates significant difference between clusters at *P* < 0.05.

When the method was applied to all salt-grown plants, sodium distribution within the root showed a clear pronounced tissue- and genotype-specificity. The representative images for one tolerant (Persia 118) and one sensitive (Iran 118) varieties for each of four measured zones (meristem, transition, elongation, and mature zone) are shown in Figure 6, and the average amounts of Na^+^ in cell vacuole and cytosol in each variety are quantified in Figures 7–10.

### Root meristem

Significantly higher quantities of Na^+^ were accumulated in the cytosol of meristematic cells in a tolerant compared with sensitive cluster (Figure 7). Here, cytosolic Na^+^ intensity ranged from the highest 179.9 ± 7.7 (in tolerant Westonia) to the lowest 20.3 ± 1.2 (in sensitive Belgrade 3), declining in a sequence Westonia > Cranbrook > Persia 118 > Iran 118 > 340 > Belgrade 3 (Figure 7A). Overall, the amount of Na^+^ stored in the cytosol of cells in the meristem region of the tolerant cluster was 4.3-fold higher compared with the sensitive cluster (159.2 ± 17.9 vs. 37.0 ± 8.4; *P* < 0.01; Figure 7C) and also significantly (*P* < 0.05) higher than vacuolar Na^+^ content (Figures 7A,B; Supplementary Figure S3). At the same time, vacuolar Na^+^ intensity in root meristem zone ranged from the highest 108.9 ± 4.4 (in sensitive Iran 118) to the lowest 56.7 ± 4.9 (in tolerant Persia 118), declining in a sequence Iran 118 > 340 > Cranbrook > Westonia > Belgrade 3 > Persia 118 (Figure 7B; Supplementary Figure S3). Overall, no significant (at *P* < 0.05 level) difference was found in vacuolar Na^+^ in meristem cell vacuoles among two contrasting clusters (Figure 7D). Furthermore, a significant (*P* = 0.05) negative correlative relationship (*r*^2^ = 0.66) was found between cytosolic Na^+^ intensity in root meristem zone and salinity-induced damage index (Supplementary Figure S4A), while a weak positive correlation (*r*^2^ = 0.22; *P* > 0.05) was found between the vacuolar Na^+^ intensity in root meristem zone and the damage index (Supplementary Figure S4E).

### Transition zone

Vacuolar Na^+^ intensity in the root transition zone was higher than cytosolic Na^+^ intensity, in both tolerant and sensitive clusters (illustrated in Figures 6E,G and quantified in Figure 8). Belgrade 3 showed the lowest (37.6 ± 2.3) cytosolic Na^+^ intensity in root transition zones, while Cranbrook showed the highest (227.7 ± 5.0) (Figure 8A). Highest vacuolar Na^+^ intensity was reported for Persia 118 (209.5 ± 6.2), and the lowest for Belgrade 3 (111.9 ± 5.8) (Figure 8B). Overall, no clear trend was observed in Na^+^ distribution between cytosol and vacuole in the root transition zone. As a result, no significant difference in either cytosolic nor vacuolar Na^+^ intensity was found between salt tolerant and sensitive clusters here (Figures 8C,D). Only very modest (*r*^2^ = 0.33; *P* = 0.23) negative correlation was found between cytosolic Na^+^ intensity and salt damage index (Supplementary Figure S4B), while no correlation was found between vacuolar Na^+^ intensity in root transition zone and damage index (*r*^2^ = 0.06, Supplementary Figure S4F).

### Elongation zone

Cells in root elongation zone had slightly higher amounts of Na^+^ in the cytosol compared with vacuoles (Figures 6I,K), both in tolerant and sensitive clusters (Figure 9). Belgrade 3 showed the lowest cytosolic Na^+^ intensity (68.4 ± 4.7), while the highest values were reported for Persia 118 (248.9 ± 2.0) (Figure 9A). Vacuolar Na^+^ intensity was the highest in a tolerant Westonia (250.4 ± 1.7) and the lowest in a sensitive variety 340 (145.4 ± 3.3) (Figure 9B). Overall, no clear trends were observed in Na^+^ distribution between cytosol and vacuole in root elongation zone in six varieties (Figure 9), and no significant difference in either cytosolic or vacuolar Na^+^ intensity was found between two contrasting clusters (Figures 9C,D). No significant (at *P* < 0.05 level) correlation was found between the damage index and cytosolic Na^+^ intensity in root elongation zone (*r*^2^ = 0.02, Supplementary Figure S4C), as well as for vacuolar Na^+^ intensity (*r*^2^ = 0.13; *P* = 0.48) (Supplementary Figure S4G).

### Mature zone

Cytosolic Na^+^ in root mature zone was significantly lower in salt-tolerant compared with salt-sensitive cluster while for vacuolar Na^+^ this trend was inverse (Figures 6M,O, 10). Another important trend was that the overall amount of Na^+^ accumulated in the cytosol was much less compared with any other zone (Figure 6). Cytosolic Na^+^ intensity in root mature zone was highest in sensitive Iran 118 (99.6 ± 15.1) and lowest in tolerant Persia 118 (13.1 ± 3.5) (Figure 10A). Salt-tolerant cultivar Westonia had the highest vacuolar Na^+^ intensity (149.0 ± 19.0), while salt-sensitive Belgrade 3 had the lowest (7.6 ± 1.3) (Figure 10B). On average, cytosolic Na^+^ intensity in mature root zone was three-fold lower in tolerant (25.4 ± 7.7) than in sensitive (73.0 ± 14.6) variety (Figure 10C; significant at *P* < 0.05). At the same time, vacuolar Na^+^ intensity in mature root zone in tolerant group was eight-fold higher compared with sensitive group (111.4 ± 22.2 vs. 13.9 ± 4.3; significant at *P* < 0.01; Figure 10D). Overall, a positive correlation (*r*^2^ = 0.57; *P* = 0.08) was found between plant damage index and cytosolic Na^+^ intensity in mature root zone (Supplementary Figure S4D), while for vacuolar Na^+^ intensity this correlation was negative (*r*^2^ = 0.59; *P* = 0.08; Supplementary Figure S4H).

## Discussion

### Vacuolar Na^+^ sequestration in mature root zone but not root apex correlates with salinity tolerance in bread wheat

For glycophytes such as bread wheat, Na^+^ is not considered to be an essential nutrient (Maathuis, 2014) and, when present in excessive quantities in soil, leads to cytosolic Na^+^ toxicity, and impairs plant growth. Maintenance of the optimal cytosolic Na^+^ level under saline conditions requires effective exclusion of Na^+^ from the cytosol, either back to external media, or into vacuole (Maathuis and Amtmann, 1999; Blumwald, 2000).

In the present work, we have investigated Na^+^ distribution between the cytosol and the vacuole in four different root zones in contrasting bread wheat varieties exposed to salinity stress. Surprisingly, vacuolar Na^+^ sequestration was correlated positively with salinity tolerance *only* in mature root zone. In contrast, no significant difference in vacuolar Na^+^ sequestration patterns was found between salt tolerant and sensitive clusters in either of other three zones: transition (Figures 8B,D), elongation (Figures 9C,D), or meristem (Figures 7C,D). At the same time, significantly lower cytosolic Na^+^ intensity was found in salt tolerant compared with sensitive cluster in mature zone (Figures 10A,B). Taken together these results suggest that the ability of mature root cell vacuoles to sequester excessive Na^+^ is one of the key determinants of salinity tolerance in bread wheat.

It should be noted that, given the non-linearity of the calibration curve (Figure 4), the fluorescent intensities measured might not linearly relate to the absolute sodium concentrations in plant tissues, so some caution is needed while interpreting the presented data in quantitative terms.

**Figure 11:**
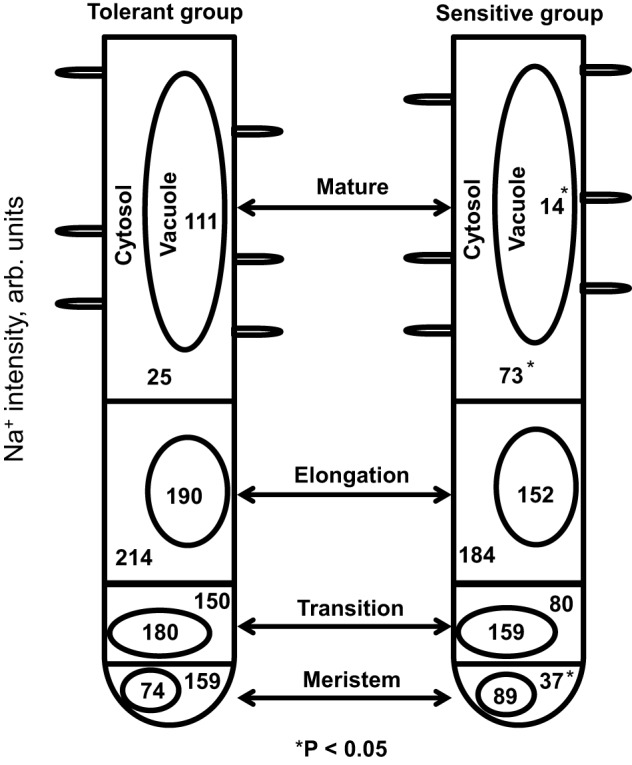
**A summary of Na^+^ distribution between various intracellular compartments and functionally different root zones in bread wheat**. Numbers represent mean pooled values for CoroNa Green fluorescence intensity (arbitrary units) for salt-sensitive and salt-tolerant clusters.

We have used term “surprisingly” above because root apex is a house for most metabolically active cells and, as such, has often considered as a potential target for many abiotic stresses such as aluminium toxicity (Doncheva et al., 2005), oxidative stress (Demidchik et al., 2007), and heavy metal toxicity (Halušková et al., 2009). It was also unexpected as SOS1 Na^+^/H^+^ exchangers that remove Na^+^ from uptake are believed to be expressed predominantly in the root apex (Shi et al., 2002). Given the fact that the functional expression of SOS1 exchangers was always considered as an important component of salinity tolerance trait (Zhu, 2003; Apse and Blumwald, 2007; Olías et al., 2009; Oh et al., 2009), the fact that cytosolic Na^+^ levels in root meristem of tolerant group was six-fold higher compared with mature zone (159.2 ± 17.9 vs. 25.4 ± 7.7, respectively; Figure 11) was unexpected.

On the other hand, mature root zone represents a major bulk of the root and, thus, has to deal with the largest quantities of accumulated Na^+^. This zone also has fully expanded cells, with large and well-formed vacuoles, while in meristematic or transition zones cells are much smaller and with small vacuoles (Verbelen et al., 2006). Thus, superior Na^+^ sequestration ability in mature root zone make sense from both anatomical and physiological points of view. Another aspect to be considered is a need to maintain cell turgor pressure under hyperosmotic conditions caused by salinity. Indeed, cytosolic Na^+^ intensity was *always* higher in elongation zone compared with all other zones in each variety studied; and so was the vacuolar Na^+^ intensity (Figure 11). It may be suggested therefore that in this zone Na^+^ might be utilized by roots as a cheap osmoticum to maintain turgor pressure and enable cell expansion. Consistent with this idea are findings that in maize root apical zone, the estimated turgor potential showed only a small decline although salt shock caused a rapid decrease in root water and solute potentials in the major bulk of the root (Rodríguez et al., 1997).

Na^+^ sequestration into vacuole is believed to be mediated by the tonoplast NHX Na^+^/H^+^ antiporters (Apse et al., 1999). Indeed, salt tolerant bread wheat variety showed higher expression of *TaNHX* in roots (Saqib et al., 2005) and also higher vacuolar Na^+^ sequestration in root mature zone compared with the sensitive one (Cuin et al., 2011). Similarly, relative expression of *ZmNHX* increased proportionally with increasing external NaCl concentration in maize in bred line roots (Zörb et al., 2005). Also, transgenic tobacco lines with *TNHXS1* (wheat Na^+^/H^+^ vacuolar antiporter gene) had greater Na^+^/H^+^ antiporter activity in root tonoplast vesicles and showed higher salt tolerance than the wild type (Gouiaa et al., 2012). Previous studies in our laboratory highlighted the importance of K^+^ retention in mature root zone of various species (Chen et al., 2005, 2007a; Cuin et al., 2008; Smethurst et al., 2008). Here we provide the evidence that the vacuolar Na^+^ sequestration in this zone correlates with salinity tolerance. Taken together, cytosolic K^+^ retention and Na^+^ sequestration represent two major components of the tissue tolerance mechanism. Targeting these traits may be a promising way to improve salinity tolerance in bread wheat.

### Root meristem zone as a salt sensor?

How plants sense Na^+^ to trigger the following signaling cascades to cope with the salt stress is a fundamental question which still need to be studied and clarified. In animals, Na^+^ sensing mechanism appears to consist mostly of specific Na^+^ selective ion channels and other Na^+^ transporters; however, so far no Na^+^ selective ion channels have been identified in plants (Maathuis, 2014). Displacement of Ca^2+^ by Na^+^ from the plasmalemma of root cells was proposed as a primary response to salt stress (Cramer et al., 1985), but it has been deemed to be of a minor importance later (Kinraide, 1999). Plasma-membrane based SOS1 Na^+^/H^+^ antiporter was also suggested as a potential salt sensor (Zhu, 2003) but the explicit evidence is still lacking. Histidine kinases (such as Hik16/Hik41 in cyanobacterium, Marin et al., 2003; or AHK1/ATHK1 in Arabidopsis, Shinozaki and Yamaguchi-Shinozaki, 2000; Tran et al., 2007) were also named as potential NaCl- and/or osmo-sensors.

Generally, root is the first organ that perceives the salt stress signal. The transition zone in root apex has been suggested as a signaling-response nexus in the root (Baluška et al., 2010). This unique zone provides the root apices with an effective mechanism to reorient growth in response to many stimuli such as salinity, gravity, temperature, moisture, oxygen availability, electric fields, and heavy metals (Verbelen et al., 2006). In our investigation, both cytosolic and vacuolar Na^+^ intensities in root transition zone showed no significant difference between salt tolerant and sensitive clusters (Figures 8C,D); no significant correlation was also found between cytosolic or vacuolar Na^+^ intensities in this zone and plant damage index (Supplementary Figures S4B,F). Thus, it appears that root transition zone is *not* the main signaling-response nexus to salt stress, at least in bread wheat.

Salt stress causes nuclear and DNA degradation in root meristematic cells (Katsuhara and Kawasaki, 1996; Liu et al., 2000; Richardson et al., 2001), and NaCl-induced nuclear DNA fragmentation in the root meristem zone was higher in *sos1* mutant (lacking the ability to remove Na^+^ to external media) compared with *Arabidopsis* wild type, when grown under saline conditions (Huh et al., 2002). From this point of view, one would expect that salt-tolerant varieties would maintain lower cytosolic Na^+^ intensity in meristem cells. This was not the case in our study (Figure 11). On the contrary, salt tolerant group showed 4.3-fold higher cytosolic Na^+^ intensity (159.2 ± 17.9 vs. 37.0 ± 8.4, respectively; Figure 11) than the sensitive cluster. At the same time, vacuolar Na^+^ intensity was not significantly different between the groups (Figure 7D). It is tempting to suggest that higher cytosolic Na^+^ intensity in salt tolerant cluster in the meristematic zone might be important to effectively convey or regulate signals during salt stress to other root zones even shoot after perceiving external salt stress. Hence, we suggest that, in addition to its role in cell division, root zone meristem also participates in, or executes, a role of the salt sensor. The specific details of this signaling mechanism should be revealed in further studies.

### Evaluating salinity tolerance by screening vacuolar Na^+^ sequestration via LSCM technique

In addition to physiological and genetic complexity of the salinity tolerance trait, the progress in breeding was also hampered by the lack of convenient screening techniques. While agronomical (e.g., biomass/yield, plant survival, or leaf injury; Munns and James, 2003; Colmer et al., 2005) and biochemical (e.g., antioxidant activity or compatible solutes content; Ashraf and Harris, 2004) markers are convenient as rapid screening tools, they are not directly linked with major physiological mechanisms conferring salinity tolerance. Thus, the demand for a technique that is, on one hand, convenient enough to be used for the high throughput screening and, on another hand, was directly linked to a specific physiological trait in question, remains high. We believe that using CoroNa Green imaging may suit this purpose.

Na^+^ measurement by CoroNa Green dye confocal imaging was firstly conducted in animals (Meier et al., 2006). It was then adopted by researchers to visualize Na^+^ distribution in both root (Park et al., 2009; Oh et al., 2009, 2010; Li et al., 2012) and leaf (Bonales-Alatorre et al., 2013a,b) tissues under saline conditions. The present work narrows down salinity tolerance trait to vacuolar Na^+^ sequestration in merely one specific root zone (Figures 10, 11) and shows a high (*r*^2^ = 0.59, Supplementary Figure S4H) prognostic value as a screening tool. In practical terms, imaging of one specific zone of a root takes only a few minutes, in addition to ~2 h required for staining. Thus, quantifying Na^+^ fluorescent intensity in 100–120 roots per day by one operator is a realistic task. From our experience, 6–8 biological replicates are sufficient to get consistent results and eliminate out layers. Thus, even at the current stage, screening 15–20 genotypes per day may be feasible. The next practical step should be creating a DH population between Westonia and Belgrade 3 (varieties with highest and lowest vacuolar Na^+^ sequestration ability), and then screening this DH population to determine QTL(s) associated with such sequestration ability. This work is next on agenda in our laboratories.

### Conflict of interest statement

The authors declare that the research was conducted in the absence of any commercial or financial relationships that could be construed as a potential conflict of interest.

## Supplementary material

Supplementary Figure S1 **Quantifying salinity tolerance in bread wheat by the damage index**. The extent of salinity damage to plants was quantified on 0 (no visual symptoms of stress) to 10 (all plants are dead) scoring scale. Two examples for tolerant variety Persia 118 **(A)** and sensitive variety 340 **(B)** are shown. **(C)** A correlation between damage index and shoot fresh weight in six bread wheat treated with 300 mM NaCl for about 5 weeks. Each point represents a variety.

Supplementary Figure S2 **Homogeneity of CoroNa Green fluorescence signal between various cell layers**. **(A,C)** Representative (one of four) images of CoroNa Green fluorescence in the root apex of bread wheat cultivar Belgrade 3 taken at different focal depth (**A**, top cell layer; **C**, fifth cell layer; ~80 μm deeper inside the root). **(B,D)** Respective light images. **(E,F)** Mean fluorescence intensity values in cytosol **(E)** and vacuole **(F)** of cells in the first (shown in **A**) and fifth (shown in **C**) cell layers. Mean ± SE (*n* = 20–24). The difference between cell layers (different Z-plains) is not significant at *P* < 0.05, neither in vacuole nor in the cytosol.

Supplementary Figure S3 **Na^+^ distribution between the cytosol and the vacuole in meristematic zone of wheat root**. A representative images of the root meristem loaded with Corona Green AM is shown for salt-tolerant cultivar Persia 118 and salt-sensitive cultivar Iran 118. As one can see, in tolerant variety most of the Na^+^ is located in the cytosol while vacuoles are dark and show not much fluorescent signal. The opposite is true for salt-sensitive genotype

Supplementary Figure S4 **Correlation between salinity stress tolerance and Na^+^ distribution in cytosol and vacuole in different root zones**. Correlation between salinity stress tolerance (quantified as a damage index) and cytosolic **(A–D)** and vacuolar **(E–H)** Na^+^ intensities in four functional root zones. Each point represents a variety.
