# Supplementary figures and images for: Linking salinity stress tolerance with tissue-specific Na+ sequestration in wheat roots
Source: Front Plant Sci. 2015 Feb 20;6:71. doi: 10.3389/fpls.2015.00071 (PMC4335180; doi:10.3389/fpls.2015.00071)

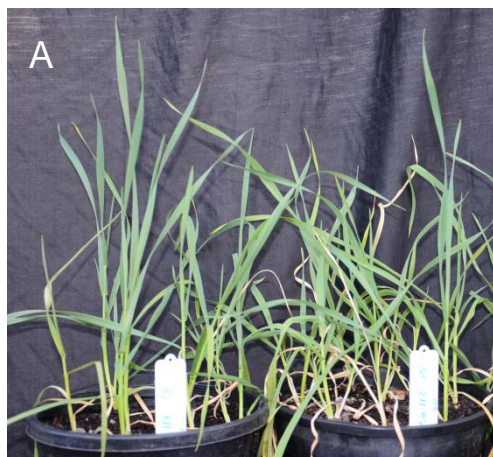

Persia 118  
(tolerant)

Score: 2.3

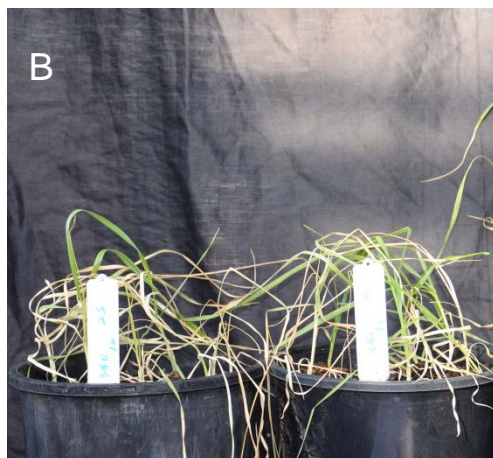

Variety 340  
(sensitive)

Score: 5.5

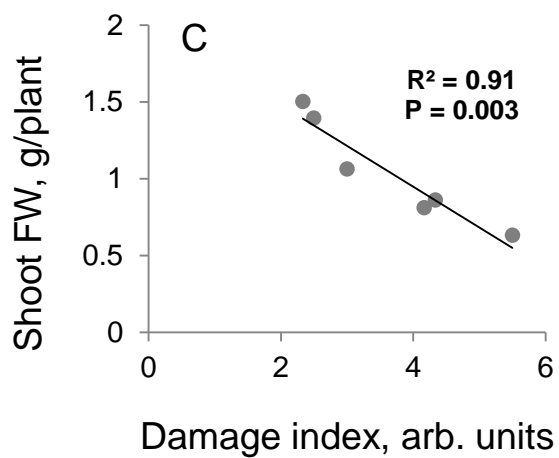

Supplement: Supplementary Figure S1 — Quantifying salinity tolerance in bread wheat by the damage index. The extent of salinity damage to plants was quantified on 0 (no visual symptoms of stress) to 10 (all plants are dead) scoring scale. Two examples for tolerant variety Persia 118 (A) and sensitive variety 340 (B) are shown. (C) A correlation between damage index and shoot fresh weight in six bread wheat treated with 300 mM NaCl for about 5 weeks. Each point represents a variety. [file Image1.PDF]

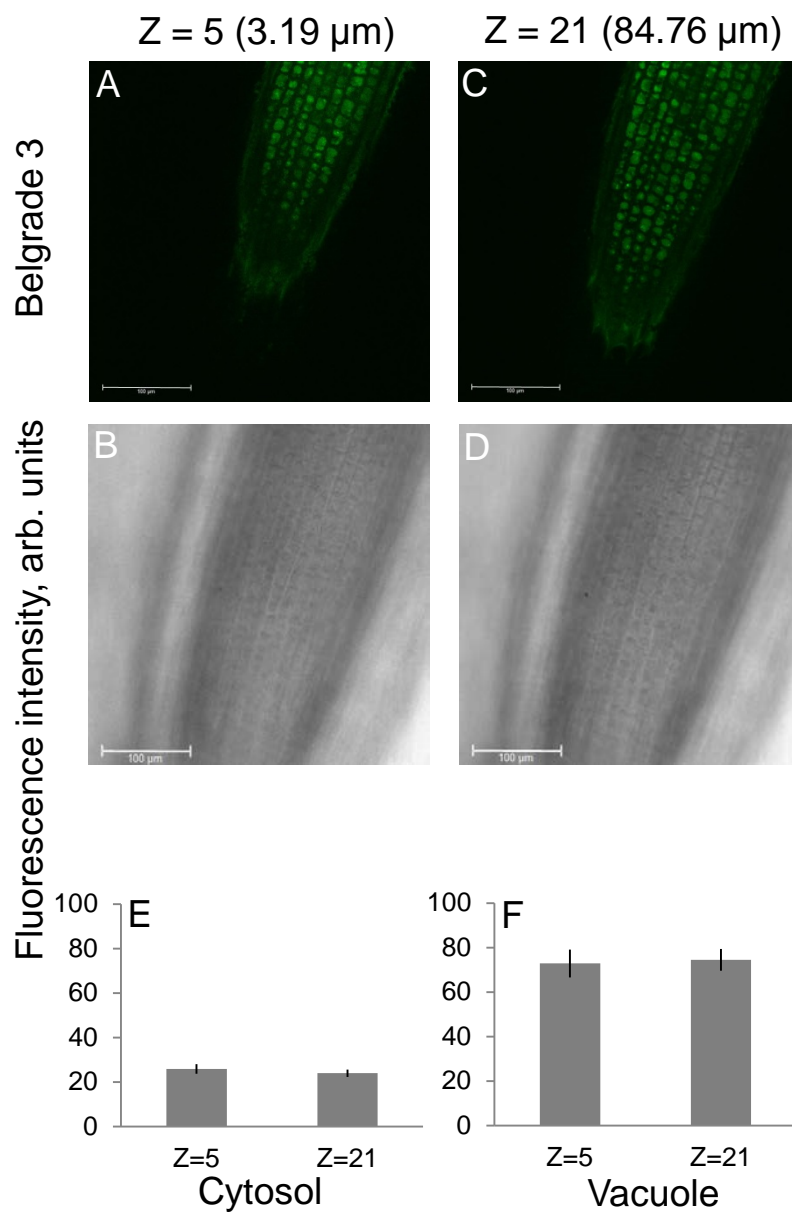

Supplement: Supplementary Figure S2 — Homogeneity of CoroNa Green fluorescence signal between various cell layers. (A,C) Representative (one of four) images of CoroNa Green fluorescence in the root apex of bread wheat cultivar Belgrade 3 taken at different focal depth (A, top cell layer; C, fifth cell layer; ~80 μm deeper inside the root). (B,D) Respective light images. (E,F) Mean fluorescence intensity values in cytosol (E) and vacuole (F) of cells in the first (shown in A) and fifth (shown in C) cell layers. Mean ± SE (n = 20–24). The difference between cell layers (different Z-plains) is not significant at P < 0.05, neither in vacuole nor in the cytosol. [file Image2.PDF]

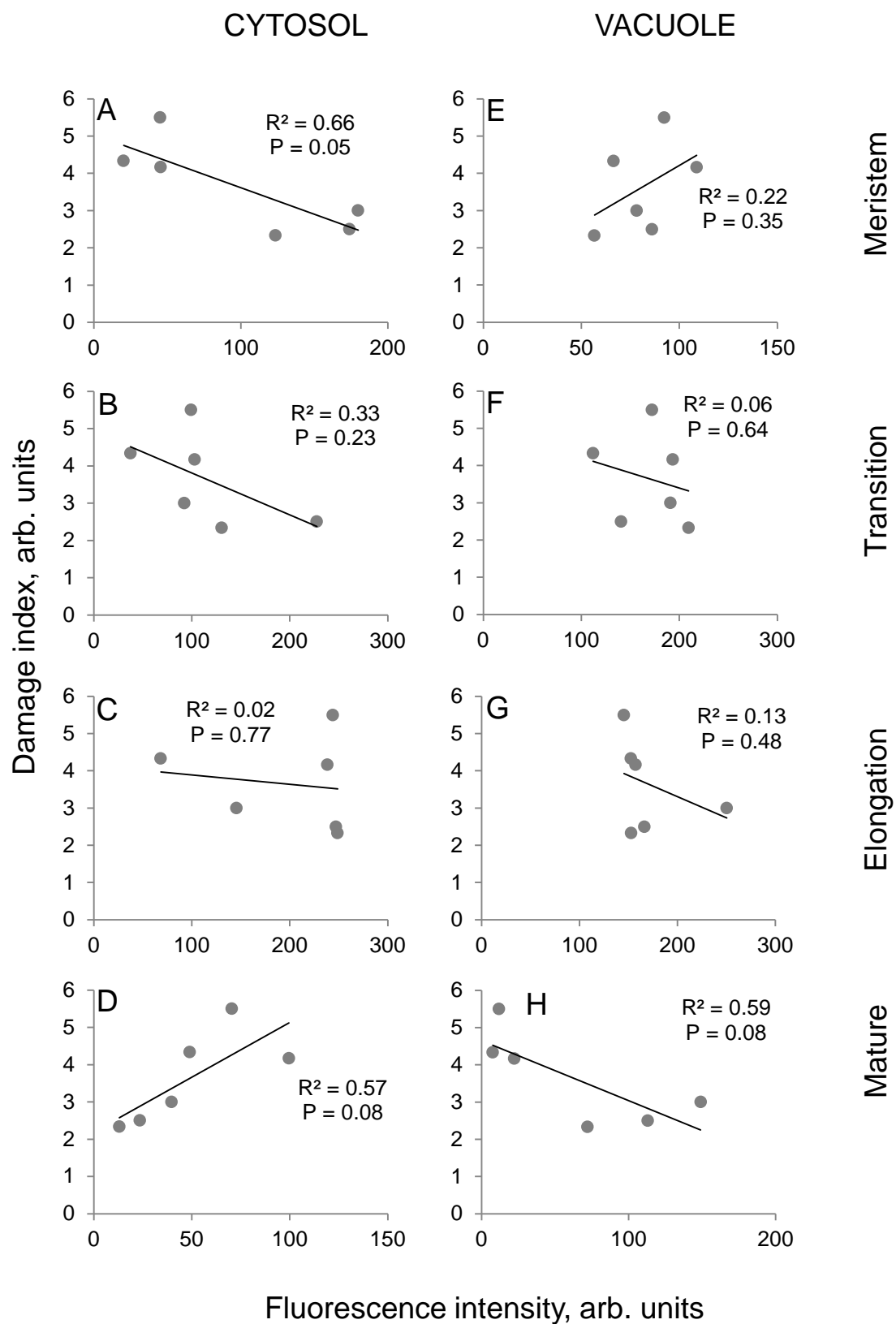

Supplement: Supplementary Figure S4 — Correlation between salinity stress tolerance and Na+ distribution in cytosol and vacuole in different root zones. Correlation between salinity stress tolerance (quantified as a damage index) and cytosolic (A–D) and vacuolar (E–H) Na+ intensities in four functional root zones. Each point represents a variety. [file Image4.PDF]
