# Supplementary material for: Linking salinity stress tolerance with tissue-specific Na+ sequestration in wheat roots
Source: Front Plant Sci. 2015 Feb 20;6:71. doi: 10.3389/fpls.2015.00071 (PMC4335180; doi:10.3389/fpls.2015.00071)
Supplement: Supplementary Figure S3 — Na+ distribution between the cytosol and the vacuole in meristematic zone of wheat root. A representative images of the root meristem loaded with Corona Green AM is shown for salt-tolerant cultivar Persia 118 and salt-sensitive cultivar Iran 118. As one can see, in tolerant variety most of the Na+ is located in the cytosol while vacuoles are dark and show not much fluorescent signal. The opposite is true for salt-sensitive genotype [file Image3.PDF]

Suppl. Fig S3

Salt-tolerant variety	Salt-sensitive variety
Persia 118	Iran 118

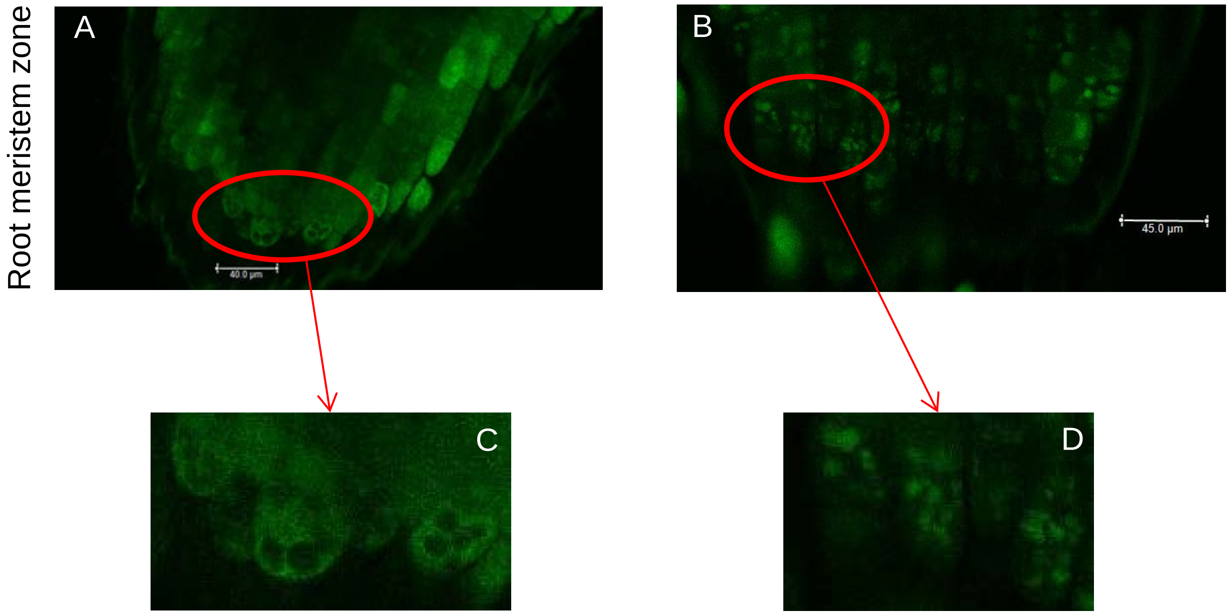

Fluorescent Na^{+} signal is lower in multiple vacuoles (dark ovals) compared with cytosol

Higher Na^{+} intensity in vacuoles compared with cytosol
